# Therapeutic Notes

**Published:** 1914-09

**Authors:** J. M. Fortescue-Brickdale


### Therapeutic Notes.

### ANIMAL EXTRACTS IN THERAPEUTICS.

THIS subject was recently discussed at the section of Pharmacology at the British Medical Association Meeting at Aberdeen. Thyroid extract was not included in the discussion, though allusions to it occasionally crept in, as it was thought that the great mass of evidence now accumulated on the action of this substance was too large to be dealt with, unless a whole sitting could be devoted to it alone.

Professor Noël Paton, who opened the discussion, pointed out the many difficulties and fallacies which surround the interpretation of experimental and clinical data on the action of animal extracts, and his paper, when it appears, should be carefully read by clinicians, as it is a valuable warning to all who may be led to draw too definite or far-reaching conclusions from their own observations, or from those which appear in the already extensive literature. For his task Professor Noël Paton was admirably fitted, as he has recently devoted considerable attention to the subject, and his conclusions, coming from a pure physiologist, are less likely to be biassed than those which are drawn by pharmacologists or physicians. Among the many difficulties and fallacies which may arise he called attention to the following: (1) Nucleo-proteins occurring in animal extracts may be missed in the process of extraction, and their active part in contributing to the physiological effect overlooked; (2) early putrefaction may produce bodies (such as choline or the putrefactive amines) with distinct pharmacological action, which may be attributed to the specific "internal secretion" of the organ; (3) dosage may introduce much variation into the action of an animal extract, as it is well known to do in the case of vegetable drugs; (4) the so-called "method of antagonism" is very difficult to employ in demonstrating the action of an internal secretion.

Again, speaking generally, the mode and place of action of an internal secretion is an extraordinarily difficult thing to determine. Thus adrenalin cannot produce glycosuria by its action on the pancreas, as it is effective after that organ is removed. Its action must be peripheral, as it occurs after section of the splanchnics. The liver has been suggested as the part on which it acts, but this view is also difficult to elucidate. Apparently the action of internal secretions is specific, and not merely a general metabolic effect, but considerable variations occur in the specificity owing to differences in the physiological sensitiveness of the same organ, both at different periods of its development and as it occurs in different animals. Professor Noël Paton considers that the specificity of extracts has been much exaggerated, and condemns *in toto* the use of advertised mixed extracts, which he characterises as the worst form of polypharmacy. He also objects to the word "hormone" as a general term for the active principles of internal secretions. Hormone implies stimulation, whereas many of these principles are inhibitory.

Dr. Grünbaum, who spoke next, reinforced the very necessary words of caution as to the interpretation of results which the first speaker had uttered. Passing on to the consideration of individual extracts, he said that he had fed a number of persons between the ages of 16 and 18, whose growth was below normal, on *suprarenal cortex*, without result. The *medulla*, on the other hand was useful in three ways : (1) In the diagnosis of Addison's disease ; (2) to tide over temporary suprarenal inadequacy, as after an injection of salvarsan ; (3) continuously in large doses (40 to 60 minims three times a day) in Addison's disease. In hæmatemesis a few minims every hour for 24 hours might be useful, but at longer intervals it did no good. In hæmophilia he had found it useless. *Parathyroid extract* he had tried in two cases of paralysis agitans without result. *Pituitary extract* he found might be used to raise the blood pressure temporarily, but repeated injections were valueless. In two cases of adiposis dolorosa he had seen improvement occur with large doses, but the remedy was costly.

*Kidney extract* he did not consider of practical value, and doubted the theoretical grounds on which its employment in nephritis was advocated. *Hormonal* he had used with appreciable results in chronic constipation, but he was inclined to attribute much of its effect to suggestion, the intramuscular injection of this substance being very painful.

Dr. Hertz, dealing first with adrenalin, advocated its use in very small doses, usually ½ minim, and never more than 1½ minim, subcutaneously to relieve the spasmodic attacks in asthma. He gave details of his own case. He believed it to act as a broncho-dilator, and did not think that these small doses, even when repeated two or three times in a night, could produce atheroma. He had tried *Parathyroid extract* in ten cases of paralysis agitans, and like Dr. Grünbaum, had obtained no results. He related, however, a curious case of definite parathyroid insufficiency after the removal of a goitre, which was cured by continuous doses of the extract.

Dr. Gunn pointed out that all cases of heightened blood pressure were not due to excess of *suprarenin*. He doubted if the action of this substance varied with the dose. On the intestine it may act in one of two ways—either directly on the blood-vessels, or on the muscularis mucosæ, making it contract and close the vessels. Experimentally *pituitrin* had no stimulant action on the heart, and it was very difficult to show that it had any action on the isolated intestine. However, it might act by making the muscle more sensitive to motor nerve impulses.

J. M. FORTESCUE-BRICKDALE.

